# Development of user-friendly functional molecular markers for *VvDXS* gene conferring muscat flavor in grapevine

**DOI:** 10.1007/s11032-013-9929-6

**Published:** 2013-08-09

**Authors:** F. Emanuelli, M. Sordo, S. Lorenzi, J. Battilana, M. S. Grando

**Affiliations:** Department of Genomics and Biology of Fruit Crops, IASMA Research and Innovation Centre, Fondazione Edmund Mach, Via E. Mach 1, 38010 San Michele all’Adige, TN Italy

**Keywords:** Aroma, Breeding, DXS, Functional markers, High-resolution melting, Grapevine

## Abstract

High fruit and wine quality combined with good climatic adaptation and disease resistance are essential objectives of grape breeding. While several molecular markers are available for pyramiding resistance to fungal pathogens, molecular tools for predicting fruit composition are still scarce. Muscat flavor, caused by the accumulation of monoterpenoids in the berry, is an important target trait for breeding, sought after in both table grapes and wine. Four missense mutations in the *VvDXS* gene in grape germplasm have been shown to be tightly linked to muscat flavor. Here we present highly reproducible and breeder-friendly functional markers for each of the targeted polymorphisms developed by using either the multiplexed minisequencing SNaPshot™ method, the high-resolution melting (HRM) assay or the cleaved amplified polymorphic sequence system. A total of 242 grapevine accessions were analyzed to optimize these different genotyping methods and to provide allele-specific markers for accurate selection of muscat flavor at early stages of grape breeding programs. The HRM and the minisequencing SNaPshot multiplex assays allow for high-throughput automated screening and are suitable for large-scale breeding programs and germplasm characterization.

**Electronic supplementary material:**

The online version of this article (doi:10.1007/s11032-013-9929-6) contains supplementary material, which is available to authorized users.

The Eurasian grape (*Vitis vinifera* L.) is a valuable horticultural crop grown for wine, table grape and dry fruit production in a global vineyard surface of 7 million ha (FAOSTAT 2011). Almost all of the grapevine varieties cultivated today are thought to have arisen spontaneously rather than by deliberate breeding, and clonal selection is the most common strategy used for improvement of classical cultivars (Töpfer et al. 2011; Torregrosa et al. 2011). The first cross-breeding effort to develop disease-resistant genotypes was undertaken in the nineteenth century, following the introduction of North American phylloxera and mildews into the European vineyards. This process generated a number of valuable hybrid rootstocks that are still being used in all wine-growing countries, but it was less successful for the resistant direct producer cultivars, as the quality of the wine was compromised by so-called “hybrid off-aroma” (Alleweldt and Possingham 1988). Nevertheless, more recent crosses have been based on cultivars with higher fruit quality, including positive aroma. In particular, very distinct muscat flavor is a desired quality trait, which combined with resistance to pathogens in modern cultivars has become a key target trait in grape development programs (Reisch et al. 2012). Molecular markers are now available to combine several sources of resistance to pathogens into one genotype (Eibach et al. 2007). However, at the same time markers for quality traits that would significantly shorten the time required for fruit evaluation (at least 4–5 years after a cross), are still scarce. The muscat aroma is due to the presence of monoterpenoids which are found at considerable levels in grapes of the Muscat family, and in moderate concentrations in a few other aromatic cultivars (Ribéreau-Gayon et al. 1975; Gunata et al. 1985; Mateo and Jiménez 2000). The gene *VvDXS*, coding for a rate-limiting enzyme in the plastidial methyl-erythritol-phosphate (MEP) pathway, was found to co-localize with the major QTL (quantitative trait locus) associated with monoterpenoid accumulation in the grape berry (Lois et al. 2000; Estévez et al. 2001; Battilana et al. 2009; Duchêne et al. 2009). A single nucleotide polymorphism (SNP) within *VvDXS* (SNP1822 G > T) causes a dominant gain-of-function K284N substitution, and was found to be strongly associated with muscat-flavored genotypes (Emanuelli et al. 2010). More recently Battilana et al. (2011) functionally proved the effect of the K284N substitution. The allele 284N showed a higher in vitro activity and a stronger increase of monoterpenoid production in transgenic tobacco plants, compared to the wild-type allele 284 K. Specific non-neutral missense mutations (S272P and R306C) as well as a five-amino-acid deletion (del GVTKQ285-289) were also identified in *VvDXS* and could discriminate Muscat-like aromatic variants of important commercial cultivars, such as Chardonnay (Chardonnay musqué, SNP1784 T > C), Traminer (Gewürztraminer, SNP1982 C > T) and Chasselas (Chasselas musqué, SNP1917 A > G), respectively (Emanuelli et al. 2010).

Here, we exploited the sequence information of *VvDXS* to develop a complete set of allele-specific markers for practical breeding based on three alternative genotyping methods: a minisequencing ABI PRISM^®^ SNaPshot™ multiplex assay, a high resolution melting (HRM) assay (Wittwer et al. 2003), and cleaved amplified polymorphic sequence (CAPS) markers (Konieczny and Ausubel 1993).

A total of 242 grapevine accessions (including inter-specific hybrids) maintained in the germplasm collection (ITA362) at Fondazione Edmund Mach (FEM) were analyzed in the present study. The plants were chosen to represent non-aromatic cultivars (21), aromatic cultivars having muscat, herbaceous, foxy or special flavor (101), and seedlings resulting from self-pollination of the Muscat-type cultivar Brachetto (93). Several somatic variants and multiple accessions of the cultivars Traminer (four aromatic and four non-aromatic), Chasselas (one aromatic and seven non-aromatic) and Chardonnay (six aromatic and three non-aromatic) were also included. With the exception of Brachetto seedlings, which were still in the juvenile phase, the rest of the accessions were mature plants in full production.

Berry flavor was characterized during two growing seasons by trained tasters who scored the accessions as 1 (non-aromatic), 2 (muscat), 3 (foxy), 4 (herbaceous) or 5 (other special flavor), according to the OIV Descriptor 236 (OIV 2009). The complete list of accessions as well as their berry flavor phenotype and *VvDXS* genotype are reported in Online Resource Table S1. Genomic DNA was extracted from 20 mg of freeze-dried leaf material using the DNeasy kit (Qiagen, Hilden, Germany) according to the manufacturer’s protocol. The DNA concentration was quantified using a NanoDrop spectrophotometer ND-100 (Thermo Scientific, USA). The primers used in the present study are reported in Online Resource Table S2 and their position is displayed in Online Resource Figure S3. The gene region containing the four SNPs under investigation (1,506 bp) was amplified in all accessions using primers DXS7F and DXS8R and 800 bp of this region were sequenced in both directions using primers DXS8F and DXS8R, following the conditions described in Emanuelli et al. (2010). Sequences were processed with Sequencing Analysis software v 3.7 (Applied Biosystems) and then assembled and manually inspected with the STADEN package v1.5.3 (Bonfield et al. 1995). The target SNPs were confirmed in the heterozygous state in six Chardonnay musqué accessions (SNP1784 T > C) and in the unique accession of Chasselas musqué (SNP1917 A > G) as well as in five aromatic Gewürztraminers and in the cultivar Siegerrebe, derived from the cross Madeleine Angevine × Traminer Rot (SNP1982 C > T). Similarly to our previous survey results, no homozygotes for SNP1784CC, SNP1917GG or SNP1982TT were found in the germplasm. When considering the SNP1822 G > T, 83 muscat-flavored cultivars were heterozygous and only three cultivars were homozygous for T, while no deviation from the expected segregation ratio was observed in the S1 progeny with 48:21:24 seedlings heterozygous, homozygous for T and homozygous for G, respectively. This would exclude a putative lethal effect of the allelic state while suggesting that the under-representation of the homozygous Muscat cultivars in the germplasm might be more likely due to the dominant nature of the mutation fixed by human selection.

The minisequencing ABI PRISM SNaPshot was optimized to be used both as a single and multiplex SNP assay by adding the primers for the minisequencing reactions with a variable length tail. Amplicons generated by using primers DXS7F and DXS8R were purified with ExoSapIT (Amersham Pharmacia Biotech, Uppsala, Sweden) according to the manufacturer’s protocol. The minisequencing reaction was performed in a final volume of 10 μl that contained 1 μl of SNaPshot primers mix (0.4 μM each primer), 5 μl of SNaPshot Multiplex Ready Reaction and 4 μl of purified PCR product. A GeneAmp PCR System 9700 was used with the following thermal conditions: 25 cycles of 96 °C for 10 s, 50 °C for 5 s and 60°c for 30 s. The final products (0.5 μl) were mixed with 9.45 μl of HiDi™ formamide and 0.05 μl of GeneScan-120 LIZ size standard (Applied Biosystems) and run in an ABI PRISM 3130xl Genetic Analyzer (Applied Biosystems). The resulting data were analyzed with the software GeneScanTM v3.7 (Applied Biosystems). The single (data not shown) and multiplex profiles were in accordance; as a multiplex assay, one single PCR reaction was followed by one multiplexed minisequencing reaction, and this produced six different profiles (Fig. 1); the profile in Fig. 1a represents non-aromatic accessions, while profiles B to D in Fig. 1 are specific for Muscat-like aromatic variants of Traminer, Chasselas and Chardonnay, respectively. The profiles in Fig. 1e, f are instead unique for Muscat genotypes. Since the minisequencing primer CHS1917_25T was designed on the reverse strand, the SNP1917 A > G is indicated as T > C. HRM curve analysis represents a fast, post-PCR high-throughput method for scanning somatic sequence alterations in target genes (Do et al. 2008; Takano et al. 2008; Garritano et al. 2009; Gonzalez-Bosquet et al. 2011). Since the four missense mutations under investigation are localized next to each other, two amplicons (CM and CT, Online Resource Figure S3C) were analyzed. Primers CMf and CMr were used to generate the amplicon CM (125 bp) covering the *VvDXS* region containing SNP1784 T > C and SNP1822 G > T, whereas primers CTf and CTr were used to obtain the amplicon CT (147 bp) containing SNP1917 A > G and SNP1922 C > T. For the HRM assay, both real-time PCR and the melting step were performed through a unique reaction process with Roche LightCycler 480^®^. For SNP1822G > T the genotyping using spike-in control DNA was also performed to distinguish rare homozygotes (T/T) from common homozygotes (G/G). In brief, genomic DNAs were mixed with an equal amount of DNA (20 %) from a known major allele homozygous individual to allow formation of heteroduplexes. This strategy converts the minor allele homozygotes into heterozygotes, making them distinguishable from the major allele homozygous samples (Garritano et al. 2009). Real-time PCR was performed in a final volume of 20 μl that contained 100 ng DNA, 2 × Master Mix (10 μl) and 0.4 μM of each primer. The thermal conditions consisted of an initial denaturation at 95 °C for 10 min, 45 cycles of 95 °C for 10 s, 60 °C for 15 s and 72 °C for 15 s, followed by the high resolution melting phase at 95 °C for 1 min, 45 °C for 1 min and 65 °C for 1 s, with melting steps in which the temperature gradually increased from 65 to 95 °C. Finally, melting curves were analyzed by using the default parameters set in the LightCycler^®^ 480 Gene Scanning software. The amplicon CM (Fig. 2a) was found to discriminate Chardonnay musqué (1714 C/T) and common Muscat genotypes (heterozygotes 1822 G/T) from the non-aromatic Chardonnay and other cultivars (i.e., non-aromatic genotypes and all the accessions of Traminer and Chasselas). The analysis of amplicon CT (Fig. 2b) allowed the discrimination of Chasselas musqué (1714 C/T) and Gewürztraminer (1982 G/T) from their respective non-aromatic variants, as well as other cultivars (i.e., non-aromatic genotypes, Muscat cultivars and all the accessions of Chardonnay). The distinction of rare homozygotes (T/T) from common homozygotes (G/G) for SNP1822G > T was not possible in HRM standard conditions (Fig. 2c), because of an insufficient difference in their melting temperature (*T*_m_). A good discrimination into three genotyping groups (G/G, G/T and T/T) was instead achieved by using pre-PCR spike-in control DNA (Fig. 2d).

**Fig. 1:**
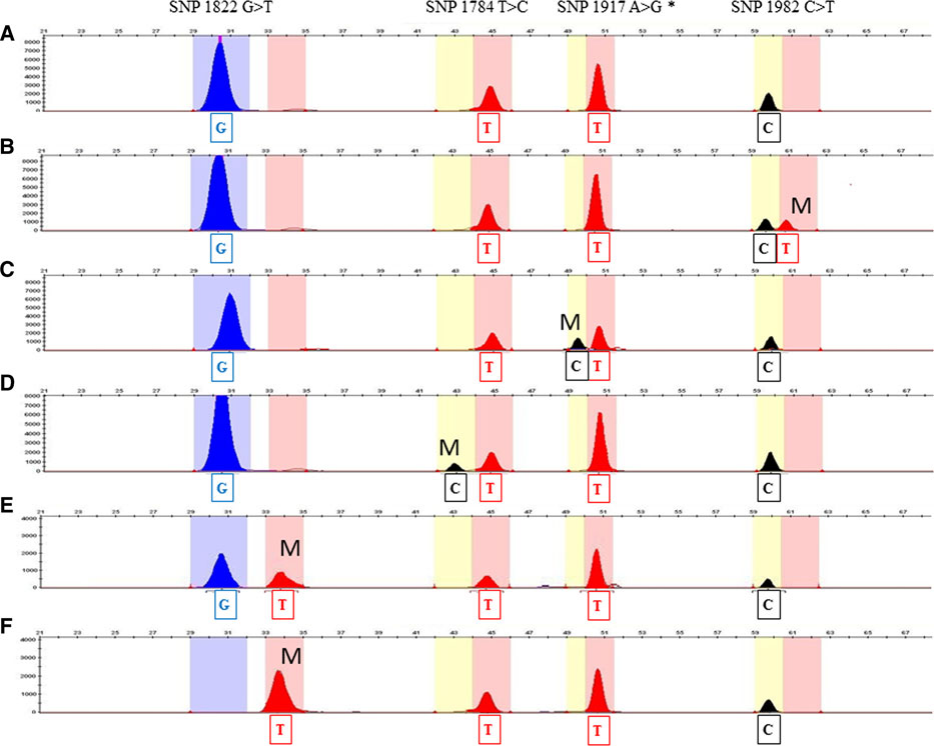
Electropherograms obtained by four-plex SNaPshot minisequencing assay. Six different multiplex profiles are visualized: electropherograms obtained from non-Muscat-like aromatic cultivars (**a**), from Muscat-like aromatic accessions of Gewürztraminer (**b**), of Chasselas musqué (**c**) and of Chardonnay musqué (**d**). Muscat cultivars presented either the electropherogram **e** (heterozygotes) or **f** (homozygotes T/T). The *x*-axis represents the size of the minisequencing products (nucleotides); the *y*-axis represents relative fluorescence units (RFUs). M = allele conferring muscat flavor in grapevines. *Since the minisequencing primer CHS1917_25T was designed on the reverse strand, the SNP1917 A > G is indicated as T > C

**Fig. 2:**
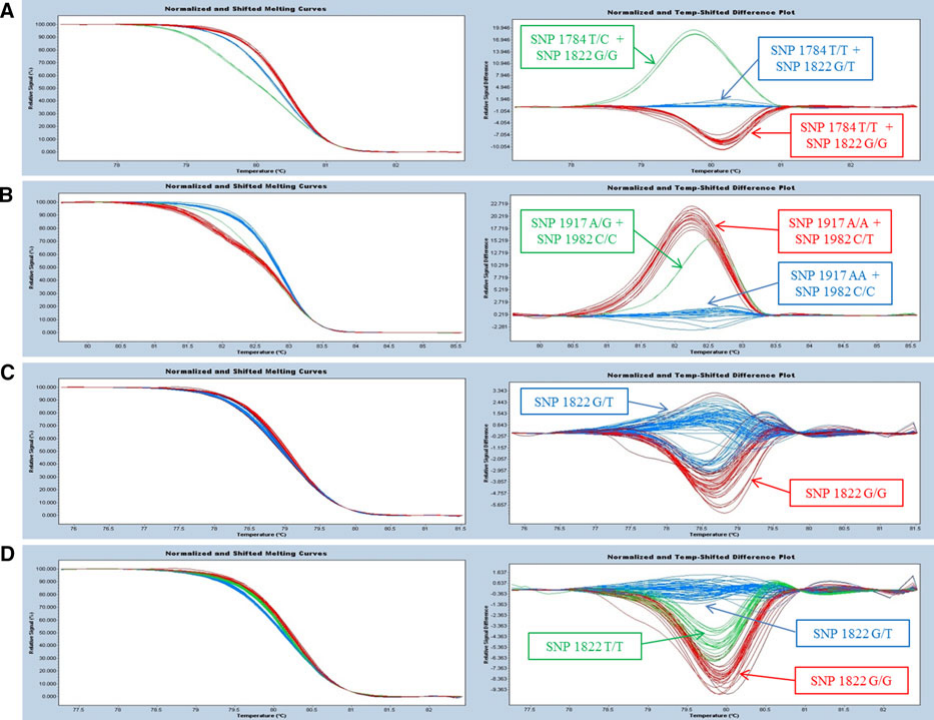
Melting profiles (normalized and shifted melting curves on the *left* and normalized and shifted difference plots on the *right*) derived by HRM analysis of **a** CM amplicon containing SNP1784 T > C and SNP1822 G > T; Chardonnay musqué samples are in *green*, Muscat genotypes (S1 seedlings heterozygotes and homozygotes TT) are in *blue* and non-aromatic clones of Chardonnay as well as other cultivars (Traminer and Chasselas clones) are in *red*; **b** CT amplicon containing SNP1917A > G and SNP1982 C > T; Chasselas musqué samples are in *green*, Muscat-like aromatic Gewürztraminer accessions are in *red* and non-aromatic clones of Chasselas and Traminer as well as other cultivars (Chardonnay clones and Brachetto S1 seedlings) are in *blue*; **c** CM amplicon used for discrimination of Brachetto S1 seedling only (SNP1822 G > T); S1 heterozygous individuals are in *blue* while S1 homozygotes G/G are in *red*; S1 homozygotes T/T are not clearly distinguished and are presented as *blue* melting curve, but showing a difference plot more similar to that of S1 homozygotes G/G (in *red*); **d** CM amplicon used for discrimination of Brachetto S1 seedling only (SNP1822 G > T) by using pre-PCR spike-in control DNA; S1 heterozygous individuals are in *blue*, while S1 homozygotes G/G are in *red* and S1 homozygotes T/T are in *green*. (Color figure online)

Aiming to describe markers that require simple protocols and relatively inexpensive laboratory equipment, CAPS markers were developed for three missense mutations, since no specific restriction enzymes could be found for the discrimination of SNP1784 T > C. Specific restriction enzymes were identified by submitting the sequence region of the amplicon (obtained by using DXS7F and DXS8R primers) to the web program NEBcutter (Vincze et al. 2003). Finally, FastDigest^®^ *Sty*I was used to detect SNP1822 G > T, FastDigest^®^ *Fau*I for SNP1917 A > G and FastDigest^®^ *Bss*SI for SNP1982 C > T (all from Fermentas) according to the manufacturer’s protocol. The restriction fragments were analyzed on 1.5 % agarose gels buffered in 0.5 × TBE (90 mM Tris–borate, 2 mM EDTA) and visualized by UV light after staining with ethidium bromide (1 μg/ml). In all three cases, the digestion profiles obtained (Fig. 3) were in accordance with the expected restriction fragment size and the restriction map inferred by NEBcutter (Online Resource Figure S3D and E). Muscat-like aromatic Traminer and Chasselas were easily discriminated from their respective non-aromatic variants as well as from the Muscat cultivars (homozygotes T/T and heterozygotes).

**Fig. 3:**
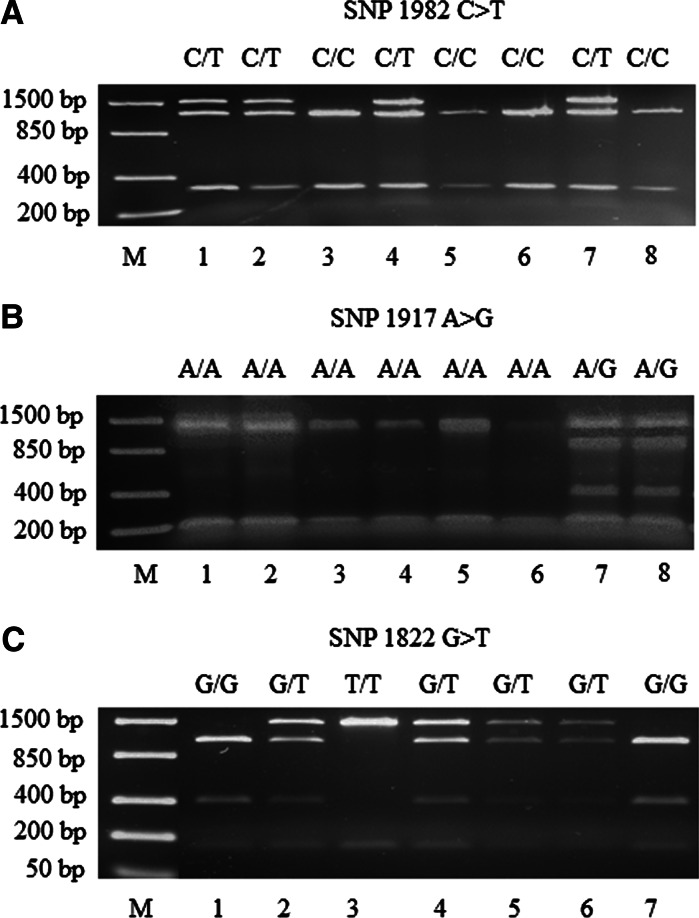
Polymorphic banding pattern of functional markers suitable for selection of muscat-flavored grapevines. **a** CAPS marker for SNP1982 C > T discriminating Muscat-like aromatic Gewürztraminer accessions (C/T, *lanes 1, 2, 4* and *7*, Gewürztraminer, Gewürztraminer 1, Ross-Taramin and Gewürztraminer ISMA 918, respectively) from neutral clones (C/C *lanes 3, 5, 6* and *8*, Albarino, Savagnin blanc, Savagnin blanc 1 and Savagnin, respectively). **b** CAPS marker for SNP1917 A > G discriminating Muscat-like aromatic Chasselas (Chasselas musqué, A/G, *lanes 7* and *8*—the same accession replicated) from neutral clones of Chasselas (A/A, *lanes 1 to 6*, Chasselas Lacinifolié, Chasselas Violet, Chasselas rose, Chasselas rouge, Chasselas apyréne and Chasselas blanc). **c** CAPS marker for SNP1822 G > T discriminating Muscat genotypes (homozygotes T/T and heterozygotes G/T*, lanes 2 to 6*, S1 progeny) from non-aromatic samples (G/G, *lanes 1* and *7*, Muskat Usbekistanskii accession 1351 and Chardonnay 130, respectively). *Lane M* contains a size marker FastRuler™ Low Range DNA Ladder (Fermentas)

In the present study, we validated specific functional molecular markers for grape aroma in a wide range of germplasm. We also optimized three different genotyping methods which may allow for a fast screening of muscat-flavored genotypes at an early stage of plant development. This can significantly reduce the cost of producing breeding lines and dramatically accelerate the breeding cycle. The development of a multiplexed assay may also be very useful for a rapid screening of germplasm collections leading to the detection of all the missense mutations at once. Our survey found only three genotypes homozygous (TT) for SNP1822 G > T, but not for the other four aromatic mutations or presenting more than one mutant allele at the same time. A previous study showed that mean concentrations of terpenols in the berries from S1 progenies of Muscat Ottonel and of Gewürztraminer were higher in plants homozygous for the aromatic allele than in heterozygotes (Duchêne et al. 2009), suggesting a potential dosage effect. Therefore, our results may lead to the possibility of breeding new varieties by combining the different mutant alleles or by evaluating aromatic homozygotes at the *VvDXS* locus. Another important application of these functional markers would be the identification of commercial aromatic clones of Gewürztraminer, Chasselas musqué and Chardonnay musqué in the nursery quality control as well as in clonal selection programs and clonal propagation. Each of the methods optimized in this paper presents specific technical advantages and can be chosen independently based on the user’s application, cost and the laboratory technologies available.

## Electronic supplementary material

Below is the link to the electronic supplementary material.

Supplementary material 1 (PDF 29 kb)

Supplementary material 2 (PDF 87 kb)

Supplementary material 3 (PDF 299 kb)

## References

[CR1] Alleweldt G, Possingham JV (1988). Progress in grapevine breeding. Theor Appl Genet.

[CR2] Battilana J, Costantini L, Emanuelli F, Sevini F, Segala C, Moser S, Velasco R, Versini G, Grando MS (2009). The 1-deoxy-D:-xylulose 5-phosphate synthase gene co-localizes with a major QTL affecting monoterpene content in grapevine. Theor Appl Genet.

[CR3] Battilana J, Emanuelli F, Gambino G, Gribaudo I, Gasperi F, Boss PK, Grando MS (2011). Functional effect of grapevine 1-deoxy-D-xylulose 5-phosphate synthase substitution K284N on Muscat flavour formation. J Exp Bot.

[CR4] Bonfield JK, Smith KF, Staden R (1995). A new DNA sequence assembly program. Nucleic Acids Res.

[CR5] Do H, Krypuy M, Mitchell PL, Fox SB, Dobrovic A (2008). High resolution melting analysis for rapid and sensitive EGFR and KRAS mutation detection in formalin fixed paraffin embedded biopsies. BMC Cancer.

[CR6] Duchêne E, Butterlin G, Claudel P, Dumas V, Jaegli N, Merdinoglu D (2009). A grapevine (Vitis vinifera L.) deoxy-d-xylulose synthase gene colocates with a major quantitative trait loci for terpenol content. Theor Appl Genet.

[CR7] Eibach R, Zyprian E, Welter L, Töpfer R (2007). The use of molecular markers for pyramiding resistance genes in grapevine breeding. Vitis.

[CR8] Emanuelli F, Battilana J, Costantini L, Le Cunff L, This P, Grando MS (2010). A candidate gene association study for muscat flavor in grapevine Vitis vinifera L. BMC Plant Biol.

[CR9] Estévez JM, Cantero A, Reindl A, Reichler S, León P (2001). 1-Deoxy-d-xylulose-5-phosphate synthase, a limiting enzyme for plastidic isoprenoid biosynthesis in plants. J Biol Chem.

[CR10] FAOSTAT (2011) FAO statistical databases, production statistics. http://faostat.fao.org/

[CR11] Garritano S, Gemignani F, Voegele C, Nguyen-Dumont T, Le Calvez-Kelm F, De Silva D, Lesueur F, Landi S, Tavtigian SV (2009). Determining the effectiveness of high resolution melting analysis for SNP genotyping and mutation scanning at the TP53 locus. BMC Genet.

[CR12] Gonzalez-Bosquet J, Calcei J, Wei JS, Garcia-Closas M, Sherman ME (2011). Detection of somatic mutations by high-resolution DNA melting (HRM) analysis in multiple cancers. PLoS ONE.

[CR13] Gunata Y, Bayonove C, Baumes R (1985). The aroma of grapes I. Extraction and determination of free and glycosidically bound fractions of some grape aroma components. J Chromatogr A.

[CR14] Konieczny A, Ausubel FM (1993). A procedure for mapping Arabidopsis mutations using co-dominant ecotype-specific PCR-based markers. Plant J.

[CR15] Lois LM, Rodríguez-ConcepciónM GallegoF, Campos N, Boronat A (2000). Carotenoid biosynthesis during tomato fruit development: regulatory role of 1-deoxy-d-xylulose 5-phosphate synthase. Plant J.

[CR16] Mateo JJ, Jiménez M (2000). Monoterpenes in grape juice and wines. J Chromatogr A.

[CR17] OIV (2009) 2nd Edition of the OIV descriptor list for grape varieties and *Vitis* species. International Organisation of Vine and Wine (OIV). http://www.oiv.int

[CR18] Reisch BI, Owens CL, Cousins PS (2012) Grape. In Badenes ML, Byrne DH (eds) Fruit breeding, handbook of plant breeding, vol 8. Springer Science + Business Media, LLC, New York, pp 225–262

[CR19] Ribéreau-Gayon P, Boiron JN, Terrier A (1975). Aroma of Muscat grape cultivars. J Agric Food Chem.

[CR20] Takano EA, Mitchell G, Fox SB, Dobrovic A (2008). Rapid detection of carriers with BRCA1 and BRCA2 mutations using high resolution melting analysis. BMC Cancer.

[CR21] Töpfer R, Hausmann L, Eibach R (2011) Molecular breeding. In: Zapater JM, Blondon AM, Kole C (eds) Genetics, genomics, and breeding of grapes. Science Publishers, New Hampshire, USA, pp 160–185

[CR22] Torregrosa L, Fernandez L, Bouquet A, Boursiquot J-M, Pelsy F, Martínez-Zapater JM (2011) Origins and consequences of somatic variation in grapevine. In: Zapater JM, Blondon AM, Kole C (eds) Genetics, genomics, and breeding of grapes. Science Publishers, New Hampshire, USA, pp 68–92

[CR23] Vincze T, Posfai J, Roberts RJ (2003) NEBcutter: a program to cleave DNA with restriction enzymes. Nucleic Acids Res 31:3688–3691. http://tools.neb.com/NEBcutter2/10.1093/nar/gkg526 PMC168933 12824395

[CR24] Wittwer CT, Reed GH, Gundry CN, Vandersteen JG, Pryor RJ (2003). High-resolution genotyping by amplicon melting analysis using LCGreen. Clin Chem.

