# Supplementary material for: Development of user-friendly functional molecular markers for VvDXS gene conferring muscat flavor in grapevine
Source: Mol Breed. 2013 Aug 9;33(1):235–41. doi: 10.1007/s11032-013-9929-6 (PMC3890582; doi:10.1007/s11032-013-9929-6)
Supplement: Supplementary file 1 — Supplementary material 1 (PDF 29 kb) [file 11032_2013_9929_MOESM1_ESM.pdf]

Online Resource Table S1: List of the samples investigated in the present study and a detailed description of accession phenotypes (berry color and berry flavor) and genotypes for the four nonsense mutations in the VvDXS gene.

N°	Use	Accession name	ID	Origin	*V.vinifera* sativa / hybrid	Berry skin color OIV 225	Berry flavor OIV 236		VvDXS gene			
							2010	2011	SNP1784	SNP1822	SNP1917	SNP1982
1	V	**Aleatico**	77	G	*V.vinifera* sativa	Rg	2	2	TT	GT	AA	CC
2	V	**Aromriesling**	145	G	*V.vinifera* sativa	B	5	5	TT	GG	AA	CC
3	V	Ascot Citronelle	153	G	*V.vinifera* sativa	B	2	2	TT	GT	AA	CC
4	V	Attila	165	G	*V.vinifera* sativa	B	2	2	TT	GT	AA	CC
5	V	AUT 120	169	G	*V.vinifera* sativa	B	2	2	TT	GT	AA	CC
6	V	Beauty Seedles	196	G	*V.vinifera* sativa	N	5	5	TT	GT	AA	CC
7	V	Beogradska Rana	204	G	*V.vinifera* sativa	B	2	2	TT	GT	AA	CC
8	V	Biczoe egon	228	G	*V.vinifera* sativa	B	2	2	TT	GT	AA	CC
9	V	Bouquetriesling	256	G	*V.vinifera* sativa	B	5	5	TT	GG	AA	CC
10	V	**Bouquettraube**	257	G	*V.vinifera* sativa	B	5	5	TT	GG	AA	CC
11	V	**Brachetto**	263	G	*V.vinifera* sativa	B	2	2	TT	GT	AA	CC
12	V	Bruni 116	273	G	*V.vinifera* sativa	B	1	1	TT	GG	AA	CC
13	V	Bruni 147	277	G	*V.vinifera* sativa	N	1	1	TT	GG	AA	CC
14	V	Bruni 360	282	G	*V.vinifera* sativa	N	2	2	TT	GT	AA	CC
15	V	Bruni 54	288	G	*V.vinifera* sativa	B	4	4	TT	GG	AA	CC
16	V	Bruni 65	289	G	*V.vinifera* sativa	B	1	1	TT	GG	AA	CC
17	V	Bruni 75	290	G	*V.vinifera* sativa	B	2	2	TT	GT	AA	CC
18	V	Cardinal	337	G	*V.vinifera* sativa	Rg	5	5	TT	GT	AA	CC
19	V	**Carmenere**	340	G	*V.vinifera* sativa	Rg	4	4	TT	GG	AA	CC
20	V	**Chaouch blanc**	405	G	*V.vinifera* sativa	B	1	1	TT	GG	AA	CC
21	V	Conegliano 213	547	G	*V.vinifera* sativa	B	2	2	TT	GT	AA	CC
22	V	Counoise	597	G	*V.vinifera* sativa	B	1	1	TT	GG	AA	CC
23	V	**Csaba gyongye**	608	G	*V.vinifera* sativa	B	2	2	TT	GT	AA	CC
24	V	Cserszegi fuszeres	609	G	*V.vinifera* sativa	Rs	2	2	TT	GT	AA	CC
25	V	Dalmasso VI-24	613	G	*V.vinifera* sativa	B	1	1	TT	GG	AA	CC
26	V	Dalmasso VIII-5	616	G	*V.vinifera* sativa	B	2	2	TT	GT	AA	CC
27	V	Dalmasso XI-8	618	G	*V.vinifera* sativa	Rg	2	2	TT	GT	AA	CC
28	V	Daranyi Ignac	629	G	*V.vinifera* sativa	B	2	2	TT	GT	AA	CC
29	V	Early Muscat	672	G	*V.vinifera* sativa	B	2	2	TT	GT	AA	CC
30	V	**Faberrebe**	712	G	*V.vinifera* sativa	B	5	5	TT	GG	AA	CC
31	V	Flame seedles	732	G	*V.vinifera* sativa	B	2	2	TT	GT	AA	CC
32	V	Gargiulo 102011	806	G	*V.vinifera* sativa	Rs	2	2	TT	GT	AA	CC
33	V	Gargiulo 102285	807	G	*V.vinifera* sativa	B	2	2	TT	GT	AA	CC
34	V	Gargiulo 102554	809	G	*V.vinifera* sativa	Rs	2	2	TT	GT	AA	CC
35	V	Gargiulo 39915	812	G	*V.vinifera* sativa	N	4	4	TT	GG	AA	CC

N	use	Accession name	ID	origin	*V.vinifera* sativa / hybrid	berry skin color OIV 225	berry flavor OIV 236		VvDXS gene			
							2010	2011	SNP1784	SNP1822	SNP1917	SNP1982
36	V	Gargiulo 87746	816	G	*V.vinifera* sativa	Rs	2	2	TT	TT	AA	CC
37	V	**Giovanna**	883	G	*V.vinifera* sativa	N	2	2	TT	GT	AA	CC
38	V	Giovanna Mathiasz (Inc, Mathiasz 210)	884	G	*V.vinifera* sativa	Rs	2	2	TT	GT	AA	CC
39	V	Huxelrebe	969	G	*V.vinifera* sativa	B	1	1	TT	GG	AA	CC
40	V	Incrocio rigotti 125	2606	G	*V.vinifera* sativa	N	4	4	TT	GG	AA	CC
41	V	Irsai Oliver	987	G	*V.vinifera* sativa	B	2	2	TT	GT	AA	CC
42	V	Italia	993	G	*V.vinifera* sativa	B	2	2	TT	GT	AA	CC
43	V	Italia	991	G	*V.vinifera* sativa	B	2	2	TT	GT	AA	CC
44	V	Izauler Czeovane	994	G	*V.vinifera* sativa	Rs	5	5	TT	GG	AA	CC
45	V	KE 5-5	1016	G	Hybrid	B	2	2	TT	GT	AA	CC
46	V	Kozma Palne muskotaly	1068	G	Hybrid	B	2	2	TT	GT	AA	CC
47	V	Lacrima	1076	G	*V.vinifera* sativa	N	5	2	TT	GT	AA	CC
48	V	Liana	1107	G	*V.vinifera* sativa	N	2	5	TT	GT	AA	CC
49	V	**Madeleine Angevine**	1124	G	*V.vinifera* sativa	B	1	1	TT	GG	AA	CC
50	V	**Madeleine royale**	1128	G	*V.vinifera* sativa	B	1	1	TT	GG	AA	CC
51	V	**Malvasia di Candia Aromatica**	1150	G	*V.vinifera* sativa	B	2	2	TT	GT	AA	CC
52	V	Malvasia del Lazio	1158	G	*V.vinifera* sativa	B	1	1	TT	GG	AA	CC
53	V	**Malvasia del Lazio**	1156	G	*V.vinifera* sativa	B	2	2	TT	GT	AA	CC
54	V	**Malvasia di Candia**	1160	G	*V.vinifera* sativa	B	1	1	TT	GG	AA	CC
55	V	Malvasia di Casorzo	1168	G	*V.vinifera* sativa	Rg	1	5	TT	GT	AA	CC
56	V	**Malvasia di Sardegna**	1170	G	*V.vinifera* sativa	Rg	4	4	TT	GG	AA	CC
57	V	Malvasia di Schierano	1174	G	*V.vinifera* sativa	Rg	2	2	TT	GT	AA	CC
58	V	Malvasia moscatel fonte grande	1185	G	*V.vinifera* sativa	Rg	1	1	TT	GG	AA	CC
59	V	Malvasia nera	1188	G	*V.vinifera* sativa	Rg	2	2	TT	TT	AA	CC
60	V	Matilde	1220	G	*V.vinifera* sativa	B	5	2	TT	GT	AA	CC
61	V	Misket Dunavski	1253	G	*V.vinifera* sativa	Rg	2	2	TT	GT	AA	CC
62	V	Molinara nera	1266	G	*V.vinifera* sativa	Rg	1	1	TT	GG	AA	CC
63	V	Morio Muskat	1285	G	*V.vinifera* sativa	B	2	2	TT	GT	AA	CC
64	V	Moscatel Rosado	1293	G	*V.vinifera* sativa	Rs	2	2	TT	GT	AA	CC
65	V	Moscatello selvatico	1295	G	*V.vinifera* sativa	B	2	2	TT	GT	AA	CC
66	V	Moscato d' Adda	1299	G	*V.vinifera* sativa	N	2	2	TT	GT	AA	CC
67	V	Moscato d' oil	1300	G	*V.vinifera* sativa	Rg	5	2	TT	GT	AA	CC
68	V	**Moscato di Terracina**	1304	G	*V.vinifera* sativa	B	5	2	TT	GT	AA	CC
69	V	**Moscato giallo**	3000	G	*V.vinifera* sativa	B	2	2	TT	GT	AA	CC
70	V	**Moscato Rosa**	1309	G	*V.vinifera* sativa	Rg	2	2	TT	GT	AA	CC

N	use	Accession name	ID	origin	*V.vinifera* sativa / hybrid	berry skin color OIV 225	berry flavor OIV 236		VvDXS gene			
							2010	2011	SNP1784	SNP1822	SNP1917	SNP1982
71	V	**Mueller Thurgau**	1315	G	*V.vinifera* sativa	B	5	5	TT	GG	AA	CC
72	V	**Muscadelle**	1317	G	*V.vinifera* sativa	B	1	5	TT	GG	AA	CC
73	V	**Muscat à petit grains rouge**	554	G	*V.vinifera* sativa	Rg	2	2	TT	GT	AA	CC
74	V	Muscat Bifère	1699	G	*V.vinifera* sativa	B	2	2	TT	GT	AA	CC
75	V	Muscat Bleu	2664	G	*V.vinifera* sativa	N	2	2	TT	GT	AA	CC
76	V	**Muscat de st. Vallier blanc**	1326	G	Hybrid	B	5	2	TT	GT	AA	CC
77	V	Muscat delecta	1327	G	*V.vinifera* sativa	B	1	5	TT	GT	AA	CC
78	V	**Muscat Fleur d'Oranger**	1328	G	*V.vinifera* sativa	B	2	2	TT	GT	AA	CC
79	V	Muscat Hamburg	1330	G	*V.vinifera* sativa	N	2	2	TT	GT	AA	CC
80	V	Muscat Hamburg	1329	G	*V.vinifera* sativa	N	2	2	TT	GT	AA	CC
81	V	**Muscat of Alexandria**	1292	G	*V.vinifera* sativa	B	2	2	TT	GT	AA	CC
82	V	**Muscat Ottonel**	1082	G	*V.vinifera* sativa	B	2	2	TT	GT	AA	CC
83	V	Muscat rouge de madere	1341	G	*V.vinifera* sativa	N	2	2	TT	GT	AA	CC
84	V	Muskat Banatski	1344	G	*V.vinifera* sativa	Rg	2	2	TT	GT	AA	CC
85	V	Muskat Cluf	1347	G	*V.vinifera* sativa	B	1	1	TT	GG	AA	CC
86	V	Muskat Diamant	1348	G	*V.vinifera* sativa	B	2	2	TT	GT	AA	CC
87	V	Muskat Ruza	1349	G	*V.vinifera* sativa	Rg	2	2	TT	GT	AA	CC
88	V	Muskat Usbekistanskii	1351	G	*V.vinifera* sativa	B	1	1	TT	GG	AA	CC
89	V	Muskat Vostochnyi	1352	G	*V.vinifera* sativa	B	2	2	TT	TT	AA	CC
90	V	Muskat Yantarnyi	1353	G	*V.vinifera* sativa	B	5	5	TT	GT	AA	CC
91	V	Muskateller Rot	1358	G	*V.vinifera* sativa	Rg	2	2	TT	GT	AA	CC
92	V	Muskatriesling	1361	G	*V.vinifera* sativa	B	2	2	TT	GT	AA	CC
93	V	Mw 54	2699	G	Hybrid	B	2	2	TT	GT	AA	CC
94	V	Mw 58	2703	G	Hybrid	B	2	2	TT	GT	AA	CC
95	V	Mw 66	2704	G	Hybrid	B	2	2	TT	GT	AA	CC
96	V	Nektar	1378	G	*V.vinifera* sativa	B	2	2	TT	GT	AA	CC
97	V	Nero	1382	G	Hybrid	N	2	2	TT	GT	AA	CC
98	V	**Perlette**	1469	G	*V.vinifera* sativa	B	2	2	TT	GT	AA	CC
99	V	Phoenix	1482	G	Hybrid	B	5	5	TT	GG	AA	CC
100	V	Piquepoul Bouschet	1597	G	*V.vinifera* sativa	B	2	2	TT	GT	AA	CC
101	V	Pirovano 338	23	G	*V.vinifera* sativa	Rs	1	5	TT	GT	AA	CC
102	V	**Pirovano 46 a = Delizia di Vaprio**	1117	G	*V.vinifera* sativa	B	2	2	TT	GT	AA	CC
103	V	Poloskei muskotaly	1613	G	Hybrid	B	2	2	TT	GT	AA	CC
104	V	Primus (Pirovano 7)	1628	G	*V.vinifera* sativa	B	2	2	TT	GT	AA	CC
105	V	Prosperi 130	1637	G	*V.vinifera* sativa	B	2	2	TT	GT	AA	CC

N	use	Accession name	ID	origin	*V.vinifera* sativa / hybrid	berry skin color OIV 225	berry flavor OIV 236		VvDXS gene			
							2010	2011	SNP1784	SNP1822	SNP1917	SNP1982
106	V	Razaki	1657	G	*V.vinifera* sativa	B	2	2	TT	GT	AA	CC
107	V	Regina dei vigneti	1667	G	*V.vinifera* sativa	B	5	2	TT	GT	AA	CC
108	V	**Regina dei vigneti**	1333	G	*V.vinifera* sativa	B	2	2	TT	GT	AA	CC
109	V	Rieslaner	1678	G	*V.vinifera* sativa	B	5	5	TT	GG	AA	CC
110	V	**Riesling Renano**	1679	G	*V.vinifera* sativa	B	5	5	TT	GG	AA	CC
111	V	**Scheurebe**	750	G	*V.vinifera* sativa	B	5	5	TT	GG	AA	CC
112	V	**Schoenburger**	1796	G	*V.vinifera* sativa	Rs	2	2	TT	GT	AA	CC
113	V	Siegerrebe	713	G	*V.vinifera* sativa	Rg	5	2	TT	GG	AA	CT
114	V	Teli muskotaly	1893	G	*V.vinifera* sativa	B	2	2	TT	GT	AA	CC
115	V	Teresita	1896	G	*V.vinifera* sativa	B	5	2	TT	GT	AA	CC
116	V	Turan	1953	G	*V.vinifera* sativa	N	2	2	TT	GT	AA	CC
117	V	Veltliner rot	1339	G	*V.vinifera* sativa	Rs	1	1	TT	GG	AA	CC
118	V	Verduzzo zani	2317	G	*V.vinifera* sativa	B	2	2	TT	GT	AA	CC
119	V	**Vernaccia di S,Gimignano**	2324	G	*V.vinifera* sativa	B	1	1	TT	GG	AA	CC
120	V	Victor (Pirovano 314)	2335	G	*V.vinifera* sativa	B	2	2	TT	GT	AA	CC
121	V	**Viogner**	2340	G	*V.vinifera* sativa	B	4	4	TT	GG	AA	CC
122	V	**Welschriesling**	911	G	*V.vinifera* sativa	B	1	1	TT	GG	AA	CC
123	V	Zala gyongye	2419	G	*V.vinifera* sativa	B	1	1	TT	GG	AA	CC
124	V	Zold veltelini	2432	G	*V.vinifera* sativa	B	1	1	TT	GG	AA	CC
125	D	PL1 F1	-	S1	*V.vinifera* sativa	nd	nd	nd	TT	TT	AA	CC
126	D	PL1 A1	-	S1	*V.vinifera* sativa	nd	nd	nd	TT	GT	AA	CC
127	D	PL1 A2	-	S1	*V.vinifera* sativa	nd	nd	nd	TT	GG	AA	CC
128	D	PL1 A3	-	S1	*V.vinifera* sativa	nd	nd	nd	TT	TT	AA	CC
129	D	PL1 A4	-	S1	*V.vinifera* sativa	nd	nd	nd	TT	GG	AA	CC
130	D	PL1 A8	-	S1	*V.vinifera* sativa	nd	nd	nd	TT	GT	AA	CC
131	D	PL1 B1	-	S1	*V.vinifera* sativa	nd	nd	nd	TT	GG	AA	CC
132	D	PL1 B12	-	S1	*V.vinifera* sativa	nd	nd	nd	TT	TT	AA	CC
133	D	PL1 B2	-	S1	*V.vinifera* sativa	nd	nd	nd	TT	GT	AA	CC
134	D	PL1 B3	-	S1	*V.vinifera* sativa	nd	nd	nd	TT	GT	AA	CC
135	D	PL1 B4	-	S1	*V.vinifera* sativa	nd	nd	nd	TT	GT	AA	CC
136	D	PL1 C1	-	S1	*V.vinifera* sativa	nd	nd	nd	TT	GG	AA	CC
137	D	PL1 C2	-	S1	*V.vinifera* sativa	nd	nd	nd	TT	GT	AA	CC
138	D	PL1 C3	-	S1	*V.vinifera* sativa	nd	nd	nd	TT	GG	AA	CC
139	D	PL1 D1	-	S1	*V.vinifera* sativa	nd	nd	nd	TT	GG	AA	CC
140	D	PL1 D2	-	S1	*V.vinifera* sativa	nd	nd	nd	TT	GG	AA	CC
141	D	PL1 D3	-	S1	*V.vinifera* sativa	nd	nd	nd	TT	GT	AA	CC

N	use	Accession name	ID	origin	*V.vinifera* sativa / hybrid	berry skin color OIV 225	berry flavor OIV 236		VvDXS gene			
							2010	2011	SNP1784	SNP1822	SNP1917	SNP1982
142	D	PL1 D4	-	S1	*V.vinifera* sativa	nd	nd	nd	TT	GT	AA	CC
143	D	PL1 D5	-	S1	*V.vinifera* sativa	nd	nd	nd	TT	GG	AA	CC
144	D	PL1 D7	-	S1	*V.vinifera* sativa	nd	nd	nd	TT	TT	AA	CC
145	D	PL1 D8	-	S1	*V.vinifera* sativa	nd	nd	nd	TT	GT	AA	CC
146	D	PL1 E1	-	S1	*V.vinifera* sativa	nd	nd	nd	TT	GT	AA	CC
147	D	PL1 E2	-	S1	*V.vinifera* sativa	nd	nd	nd	TT	GT	AA	CC
148	D	PL1 E3	-	S1	*V.vinifera* sativa	nd	nd	nd	TT	GT	AA	CC
149	D	PL1 E5	-	S1	*V.vinifera* sativa	nd	nd	nd	TT	TT	AA	CC
150	D	PL1 E6	-	S1	*V.vinifera* sativa	nd	nd	nd	TT	TT	AA	CC
151	D	PL1 E7	-	S1	*V.vinifera* sativa	nd	nd	nd	TT	GT	AA	CC
152	D	PL1 E8	-	S1	*V.vinifera* sativa	nd	nd	nd	TT	GT	AA	CC
153	D	PL1 F2	-	S1	*V.vinifera* sativa	nd	nd	nd	TT	GT	AA	CC
154	D	PL1 F3	-	S1	*V.vinifera* sativa	nd	nd	nd	TT	TT	AA	CC
155	D	PL1 F7	-	S1	*V.vinifera* sativa	nd	nd	nd	TT	GT	AA	CC
156	D	PL1 F8	-	S1	*V.vinifera* sativa	nd	nd	nd	TT	GT	AA	CC
157	D	PL1 G1	-	S1	*V.vinifera* sativa	nd	nd	nd	TT	TT	AA	CC
158	D	PL1 G2	-	S1	*V.vinifera* sativa	nd	nd	nd	TT	GG	AA	CC
159	D	PL1 G3	-	S1	*V.vinifera* sativa	nd	nd	nd	TT	GT	AA	CC
160	D	PL1 G4	-	S1	*V.vinifera* sativa	nd	nd	nd	TT	TT	AA	CC
161	D	PL1 G7	-	S1	*V.vinifera* sativa	nd	nd	nd	TT	GT	AA	CC
162	D	PL1 H1	-	S1	*V.vinifera* sativa	nd	nd	nd	TT	GT	AA	CC
163	D	PL1 H2	-	S1	*V.vinifera* sativa	nd	nd	nd	TT	GT	AA	CC
164	D	PL1 H3	-	S1	*V.vinifera* sativa	nd	nd	nd	TT	TT	AA	CC
165	D	PL1 H4	-	S1	*V.vinifera* sativa	nd	nd	nd	TT	GG	AA	CC
166	D	PL1 H5	-	S1	*V.vinifera* sativa	nd	nd	nd	TT	TT	AA	CC
167	D	PL1 H6	-	S1	*V.vinifera* sativa	nd	nd	nd	TT	TT	AA	CC
168	D	PL2 A11	-	S1	*V.vinifera* sativa	nd	nd	nd	TT	GT	AA	CC
169	D	PL2 A5	-	S1	*V.vinifera* sativa	nd	nd	nd	TT	GT	AA	CC
170	D	PL2 B12	-	S1	*V.vinifera* sativa	nd	nd	nd	TT	TT	AA	CC
171	D	PL2 C10	-	S1	*V.vinifera* sativa	nd	nd	nd	TT	GT	AA	CC
172	D	PL2 C12	-	S1	*V.vinifera* sativa	nd	nd	nd	TT	GT	AA	CC
173	D	PL2 C4	-	S1	*V.vinifera* sativa	nd	nd	nd	TT	GT	AA	CC
174	D	PL2 C5	-	S1	*V.vinifera* sativa	nd	nd	nd	TT	GG	AA	CC
175	D	PL2 C6	-	S1	*V.vinifera* sativa	nd	nd	nd	TT	GG	AA	CC
176	D	PL2 C7	-	S1	*V.vinifera* sativa	nd	nd	nd	TT	TT	AA	CC
177	D	PL2 D10	-	S1	*V.vinifera* sativa	nd	nd	nd	TT	GT	AA	CC

N	use	Accession name	ID	origin	*V.vinifera* sativa / hybrid	berry skin color OIV 225	berry flavor OIV 236		VvDXS gene			
							2010	2011	SNP1784	SNP1822	SNP1917	SNP1982
178	D	PL2 D3	-	S1	*V.vinifera* sativa	nd	nd	nd	TT	GG	AA	CC
179	D	PL2 D5	-	S1	*V.vinifera* sativa	nd	nd	nd	TT	GT	AA	CC
180	D	PL2 D6	-	S1	*V.vinifera* sativa	nd	nd	nd	TT	GG	AA	CC
181	D	PL2 D8	-	S1	*V.vinifera* sativa	nd	nd	nd	TT	GG	AA	CC
182	D	PL2 D9	-	S1	*V.vinifera* sativa	nd	nd	nd	TT	GG	AA	CC
183	D	PL2 E12	-	S1	*V.vinifera* sativa	nd	nd	nd	TT	TT	AA	CC
184	D	PL2 E3	-	S1	*V.vinifera* sativa	nd	nd	nd	TT	GG	AA	CC
185	D	PL2 E4	-	S1	*V.vinifera* sativa	nd	nd	nd	TT	GT	AA	CC
186	D	PL2 E5	-	S1	*V.vinifera* sativa	nd	nd	nd	TT	GT	AA	CC
187	D	PL2 E9	-	S1	*V.vinifera* sativa	nd	nd	nd	TT	GT	AA	CC
188	D	PL2 F11	-	S1	*V.vinifera* sativa	nd	nd	nd	TT	GT	AA	CC
189	D	PL2 G11	-	S1	*V.vinifera* sativa	nd	nd	nd	TT	GT	AA	CC
190	D	PL2 G7	-	S1	*V.vinifera* sativa	nd	nd	nd	TT	GG	AA	CC
191	D	PL2 H6	-	S1	*V.vinifera* sativa	nd	nd	nd	TT	TT	AA	CC
192	D	PL2 H7	-	S1	*V.vinifera* sativa	nd	nd	nd	TT	GG	AA	CC
193	D	PL2 H9	-	S1	*V.vinifera* sativa	nd	nd	nd	TT	TT	AA	CC
194	D	PL3 A10	-	S1	*V.vinifera* sativa	nd	nd	nd	TT	GT	AA	CC
195	D	PL3 A8	-	S1	*V.vinifera* sativa	nd	nd	nd	TT	GT	AA	CC
196	D	PL3 A9	-	S1	*V.vinifera* sativa	nd	nd	nd	TT	GG	AA	CC
197	D	PL3 B10	-	S1	*V.vinifera* sativa	nd	nd	nd	TT	GT	AA	CC
198	D	PL3 B8	-	S1	*V.vinifera* sativa	nd	nd	nd	TT	GT	AA	CC
199	D	PL3 C1	-	S1	*V.vinifera* sativa	nd	nd	nd	TT	TT	AA	CC
200	D	PL3 C10	-	S1	*V.vinifera* sativa	nd	nd	nd	TT	GG	AA	CC
201	D	PL3 C11	-	S1	*V.vinifera* sativa	nd	nd	nd	TT	GT	AA	CC
202	D	PL3 C12	-	S1	*V.vinifera* sativa	nd	nd	nd	TT	GT	AA	CC
203	D	PL3 C8	-	S1	*V.vinifera* sativa	nd	nd	nd	TT	GG	AA	CC
204	D	PL3 D1	-	S1	*V.vinifera* sativa	nd	nd	nd	TT	TT	AA	CC
205	D	PL3 D11	-	S1	*V.vinifera* sativa	nd	nd	nd	TT	GT	AA	CC
206	D	PL3 D9	-	S1	*V.vinifera* sativa	nd	nd	nd	TT	GT	AA	CC
207	D	PL3 E1	-	S1	*V.vinifera* sativa	nd	nd	nd	TT	TT	AA	CC
208	D	PL3 E9	-	S1	*V.vinifera* sativa	nd	nd	nd	TT	GG	AA	CC
209	D	PL3 F7	-	S1	*V.vinifera* sativa	nd	nd	nd	TT	GT	AA	CC
210	D	PL3 F9	-	S1	*V.vinifera* sativa	nd	nd	nd	TT	GT	AA	CC
211	D	PL3 G7	-	S1	*V.vinifera* sativa	nd	nd	nd	TT	GG	AA	CC
212	D	PL3 G8	-	S1	*V.vinifera* sativa	nd	nd	nd	TT	GT	AA	CC
213	D	PL3 G9	-	S1	*V.vinifera* sativa	nd	nd	nd	TT	GT	AA	CC

N	use	Accession name	ID	origin	*V.vinifera* sativa / hybrid	berry skin color OIV 225	berry flavor OIV 236		VvDXS gene			
							2010	2011	SNP1784	SNP1822	SNP1917	SNP1982
214	D	PL3 H10	-	S1	*V.vinifera* sativa	nd	nd	nd	TT	GT	AA	CC
215	D	PL3 H4	-	S1	*V.vinifera* sativa	nd	nd	nd	TT	TT	AA	CC
216	D	PL3 H7	-	S1	*V.vinifera* sativa	nd	nd	nd	TT	GT	AA	CC
217	D	PL3 H9	-	S1	*V.vinifera* sativa	nd	nd	nd	TT	GT	AA	CC
226	D	**Gewùrztraminer ***	864	G	*V.vinifera* sativa	Rs	5	2	TT	GG	AA	CT
227	D	**Gewùrztraminer 1 ***	867	G	*V.vinifera* sativa	Rs	5	2	TT	GG	AA	CT
236	D	Albarino *	69	G	*V.vinifera* sativa	B	1	1	TT	GG	AA	CC
239	D	**Savagnin blanc ***	1780	G	*V.vinifera* sativa	B	1	1	TT	GG	AA	CC
241	D	**Savagnin blanc 1 ***	1782	G	*V.vinifera* sativa	B	1	1	TT	GG	AA	CC
242	D	**Savagnin ***	1779	G	*V.vinifera* sativa	Rs	5	2	TT	GG	AA	CT
243	D	**Savagnin blanc 2 ***	1781	G	*V.vinifera* sativa	B	1	1	TT	GG	AA	CC
244	D	**Gewùrztraminer isma 918 ***	-	C	*V.vinifera* sativa	Rs	5	2	TT	GG	AA	CT
245	D	**Chardonnay 130 ****	-	C	*V.vinifera* sativa	B	1	1	TT	GG	AA	CC
246	D	**Chardonnay 809 ****	-	C	*V.vinifera* sativa	B	2	2	TC	GG	AA	CC
255	D	**Chardonnay ****	408	G	*V.vinifera* sativa	B	1	1	TT	GG	AA	CC
257	D	**Chardonnay Musquè ****	423	G	*V.vinifera* sativa	B	2	2	TC	GG	AA	CC
258	D	**Chardonnay Musquè ****	424	G	*V.vinifera* sativa	B	2	2	TC	GG	AA	CC
260	D	**Chardonnay Musquè ****	426	G	*V.vinifera* sativa	B	2	2	TC	GG	AA	CC
261	D	**Chardonnay Musquè ****	420	G	*V.vinifera* sativa	B	2	2	TC	GG	AA	CC
262	D	**Chardonnay Musquè ****	428	G	*V.vinifera* sativa	B	2	2	TC	GG	AA	CC
264	D	**Chardonnay rosè ****	433	G	*V.vinifera* sativa	Rs	1	1	TT	GG	AA	CC
271	D	**Chasselas Lacinifolié *****	444	G	*V.vinifera* sativa	B	1	1	TT	GG	AA	CC
272	D	**Chasselas Violet *****	452	G	*V.vinifera* sativa	Rs	1	1	TT	GG	AA	CC
275	D	**Chasselas rose *****	447	G	*V.vinifera* sativa	B	1	1	TT	GG	AA	CC
277	D	**Chasselas musqué *****	445	G	*V.vinifera* sativa	B	2	2	TT	GG	AG	CC
278	D	**Chasselas rouge *****	449	G	*V.vinifera* sativa	Rg	1	1	TT	GG	AA	CC
279	D	**Chasselas apyréne *****	440	G	*V.vinifera* sativa	B	1	1	TT	GG	AA	CC
280	D	**Chasselas blanc *****	441	G	*V.vinifera* sativa	B	1	1	TT	GG	AA	CC
282	D	**Chasselas 1 *****	438	G	*V.vinifera* sativa	B	1	1	TT	GG	AA	CC

Id = accession number; D = panel of accessions used for developing the genotyping assays, V = panel of accessions used for validating the genotyping assays; G = genotypes maintained in the germplasm collection (ITA362) at Fondazione Edmund Mach; S1 = Brachetto self-fertilized progeny; C = commercial clones; * = accessions of Traminer; ** = accessions of Chardonnay; ** = accessions of Chasselas. Names of accessions registered as synonymous to the true-to-type prime name are indicated in bold (*Vitis International Variety* Catalogue http://www.vivc.de)
