# Supplementary material for: Development of user-friendly functional molecular markers for VvDXS gene conferring muscat flavor in grapevine
Source: Mol Breed. 2013 Aug 9;33(1):235–41. doi: 10.1007/s11032-013-9929-6 (PMC3890582; doi:10.1007/s11032-013-9929-6)
Supplement: Supplementary file 2 — Supplementary material 2 (PDF 87 kb) [file 11032_2013_9929_MOESM2_ESM.pdf]

Online Resource Table S2: List of primers used for PCR, Sanger sequencing, minisequencing and HRM analysis.

Primer name	Primer sequence 5'-3'	forward / reverse	use
DXS7F	GGTTACAATCTCACCTTCTCTG	forward	PCR
DXS8R	GCTAGACAGAACAGGTAAGAT	reverse	PCR, sequencing
DXS8F	GTCATAGGTGATGGAGCCA	forward	sequencing
M1822	ATTACGAGGTTGCCAA	forward	minisequencing
CH1784_15T	[15T]AGTGCTCTTAGTAGGTTACAA	forward	minisequencing
CHS1917_25T	[25T]ATCTGTTTGGTAACGCCC	reverse	minisequencing
TRA1982_35T	[35T]CAAAAGTTGATGAATATGCT	forward	minisequencing
CMf	CCCATACCACTGTAGGA	forward	HRM
CMr	AAAGATTTATCTATTCTAAAGATCACA	reverse	HRM
CTf	TGTTATTATCCCAAAGTGAACAT	forward	HRM
CTr	AAAGTGTTGATCCAGAACCA	reverse	HRM
