# Supplementary material for: Development of user-friendly functional molecular markers for VvDXS gene conferring muscat flavor in grapevine
Source: Mol Breed. 2013 Aug 9;33(1):235–41. doi: 10.1007/s11032-013-9929-6 (PMC3890582; doi:10.1007/s11032-013-9929-6)
Supplement: Supplementary file 3 — Supplementary material 3 (PDF 299 kb) [file 11032_2013_9929_MOESM3_ESM.pdf]

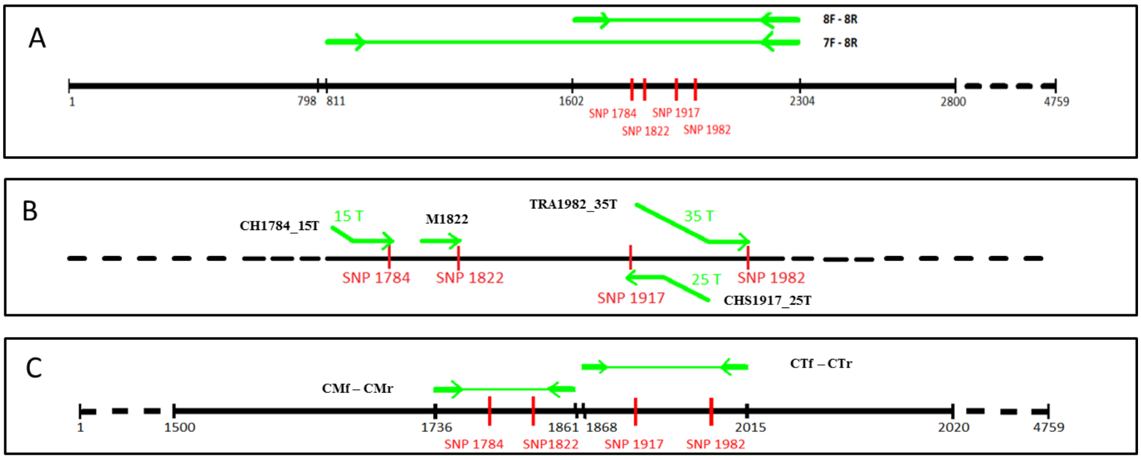

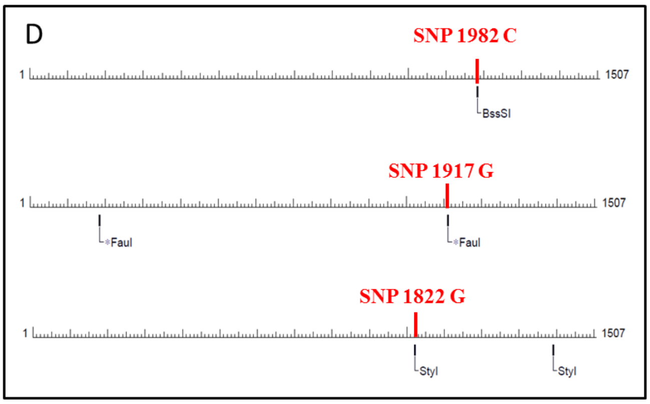

E

marker	allele	expected restriction fragments size		
SNP1982	C	1190 bp	317 bp	
	T	1507 bp		
SNP1917	A	1420 bp	187 bp	
	G	1030 bp	390 bp	187 bp
SNP1822	G	1020 bp	377 bp	110 bp
	T	1397 bp	110 bp	

Online Resource Figure S3

Primer and amplicon localization in *VvDXS* gene (A to C), restriction sites positions (D) and the expected size of bands for the CAPS markers (E).
